# Degradation Mechanism of Porous Metal-Organic Frameworks by In Situ Atomic Force Microscopy

**DOI:** 10.3390/nano11030722

**Published:** 2021-03-13

**Authors:** Ioanna Christodoulou, Tom Bourguignon, Xue Li, Gilles Patriarche, Christian Serre, Christian Marlière, Ruxandra Gref

**Affiliations:** 1 Institute of Molecular Sciences, UMR CNRS 8214, Université Paris Saclay, 91400 Orsay, France; ioanna.christodoulou@universite-paris-saclay.fr (I.C.); tom.bourguignon@universite-paris-saclay.fr (T.B.); xue.li@universite-paris-saclay.fr (X.L.); 2 Institut des Matériaux Poreux de Paris, UMR 8004, Ecole Normale Supérieure, ESPCI Paris, CNRS, PSL University, 75005 Paris, France; christian.serre@ens.psl.eu; 3 Center for Nanoscience and Nanotechnology, UMR 9001, CNRS, Université Paris Saclay, 75000 Palaiseau, France; gilles.patriarche@c2n.upsaclay.fr; 4 Laboratoire de Physique des Solides, UMR CNRS 8502, Université Paris Saclay, 91400 Orsay, France; christian.marliere@universite-paris-saclay.fr

**Keywords:** metal organic frameworks, in situ AFM, degradation, bio applications

## Abstract

In recent years, Metal-Organic Frameworks (MOFs) have attracted a growing interest for biomedical applications. The design of MOFs should take into consideration the subtle balance between stability and biodegradability. However, only few studies have focused on the MOFs’ stability in physiological media and their degradation mechanism. Here, we investigate the degradation of mesoporous iron (III) carboxylate MOFs, which are among the most employed MOFs for drug delivery, by a set of complementary methods. In situ AFM allowed monitoring with nanoscale resolution the morphological, dimensional, and mechanical properties of a series of MOFs in phosphate buffer saline and in real time. Depending on the synthetic route, the external surface presented either well-defined crystalline planes or initial defects, which influenced the degradation mechanism of the particles. Moreover, MOF stability was investigated under different pH conditions, from acidic to neutral. Interestingly, despite pronounced erosion, especially at neutral pH, the dimensions of the crystals were unchanged. It was revealed that the external surfaces of MOF crystals rapidly respond to in situ changes of the composition of the media they are in contact with. These observations are of a crucial importance for the design of nanosized MOFs for drug delivery applications.

## 1. Introduction

Metal Organic Frameworks (MOFs) are a recent class of tunable hybrid materials crafted from metal connecting points and organic bridging ligands. The nanosized MOFs (nanoMOFs) have emerged as promising candidates for biomedical applications such as drug delivery [1,2,3]. Their high and modular porosity and internal amphiphilic microenvironment allow the incorporation of a variety of drug molecules with various physicochemical properties (hydrophobic, hydrophilic, and amphiphilic) reaching high drug loadings up to 20–70 wt% and yields close to 100% [4,5]. The drugs penetrated through the nanoMOFs accessible porosity, while versatile strategies were developed to functionalize the nanoMOFs’ external surfaces with cyclodextrins, (co)polymers, lipids, or silica shells [6,7]. In recent years, the design and synthesis of MOFs for biomedical applications has attracted a growing interest. However, only few studies have focused on the MOFs’ stability in physiological media and their degradation mechanism with the exception of a few recent studies [8,9,10]. These aspects determine the biological fate of the nanoMOFs after their intravenous administration. On one hand, the nanoMOFs should present a good stability when circulating in the bloodstream, to ferry their cargo to the biological targets. On the other hand, they should also degrade to release their cargo and avoid accumulation inside the body. Therefore, the design of nanoMOFs should take into consideration the subtle balance between stability and biodegradability. This calls for a better understanding of the mechanisms involved in MOF degradation and their optimization as drug carriers.

Numerous MOF structures have been reported as candidates for biomedical applications. Zr based MOFs have been proposed as drug delivery systems. In general, these MOFs are highly porous, exhibit good chemical stability in water, and can host large quantities of drugs [5]. The UiO series (University of Oslo), the PCN-n series (Porous Coordination Network) and the NU family (Northwestern University) include large-pore Zr-based MOFs that have attracted great interest in biomedicine. Another example is the zinc imidazolate framework ZIF-8, constructed from Zn^2+^ ions and 2-methylimidazolate linkers. This material enables an on-demand release in the presence of an external stimulus such as the pH [11]. MOFs belonging to MIL-n series (MIL stands for Material of the Institute Lavoisier) have also been studied for drug delivery applications, thanks to their versatility and their biocompatibility [12]. Among the family of MOFs candidates for biomedical applications, the mesoporous iron (III) carboxylate MIL-100(Fe) accounts for one of the most studied materials for drug incorporation and release. This material has been selected here in reason of its numerous advantages for drug delivery applications: (i) Biofriendly composition (Fe is an endogenous metal and the trimesate ligand is non toxic) [13,14]; (ii) its mesoporosity, enabling the hostage of various drugs reaching high payloads and/or synergetic co-encapsulations [12,15]; (iii) its chemical stability in water; (iv) its “green” preparation [16,17]; (v) the numerous possibilities of surface modification [18,19], and (vi) its proven biodegradability and lack of toxicity both in vitro and in vivo [20,21,22].

MIL-100(Fe) nanoMOFs could host a variety of drugs including the anticancer drugs doxorubicin, gemcitabine monophosphate, or busulfan [23,24,25], anti-inflammatory drugs such as ibuprofen [26], a series of nucleoside reverse transcriptase inhibitors (NRTIs) for anti-HIV therapy [27], a combination of antibiotics [15], and very recently two co-encapsulated drugs used in skin disorders azelaic acid as antibiotic, and nicotinamide as anti-inflammatory drug) [28]. MIL-100(Fe) nanoMOFs were shown to be stable in aqueous and ethanolic solutions, but degraded rapidly upon incubation in simulated biological media, such as PBS enriched or not with proteins, simulated intestinal fluid (SIF) supplemented or not with pancreatin and cell culture media (DMEM or RPMI), and serum, releasing an important amount of their carboxylate ligands [14,29,30]. It was shown that the degradation of MIL-100(Fe) nanoMOFs depends mainly on its interaction with specific components of the release media. More precisely, complexing ions diffuse in the matrix and coordinate with the unsaturated iron (III) sites, leading to a progressive loss of the MOF constitutive ligands (trimesic acid) [29,31]. A previous study in our group indicated that degradation of larger microparticles of MIL-100(Fe) in PBS was associated with the progressive formation of an inorganic fragile corona around the MOFs. Interestingly, the microparticles maintained their initial size and morphology despite a massive loss of their constitutive ligands [10].

In another work, it was shown that MIL-100(Fe) nanoMOFs have the tendency to reversibly aggregate depending on the pH and the ionic strength of the suspension media [24]. More precisely, the nanoMOFs remained colloidally stable in acidic conditions and experienced aggregation in neutral ones. The authors attributed this phenomenon to the changes of the ζ-potential of the particles related to their surface charge. Taking advantage of this phenomenon, these nanoMOFs were used to passively target tumors in the lungs. Immediately after administration, the nanoMOFs aggregated at the neutral pH of the blood and ended up in the lungs where they released the drug cargo to the tumor. Eventually, the nanoMOFs disassembled again and it was hypothesized that this was due to surface erosion.

In this context, it is important to better understand the degradation of MIL-100(Fe) MOFs and more particularly their surface erosion by an in situ method. Atomic Force Microscopy (AFM) is an in situ microscopic technique of choice, widely used to study the surface properties of (bio)materials. It accounts for subtle height variations at any point on the surface of the observed specimen with nanoscale resolution, as well as for the surface mechanical properties.

Here we show the application of AFM to study the surface erosion and degradation of MIL-100(Fe) MOFs, both nano- and micron-scale particles. Micron-scale particles were selected, because of their slower degradation compared to the nanoMOFs, facilitating, in this manner, the exploration of the mechanism. Moreover, the AFM technique used for this study, could be performed only with particles of larger sizes. AFM is advantageously applicable to studies in liquid phase, which mimic the interaction of the crystalline material with the degradation medium as a function of its composition and pH. PBS solution was chosen, as it is one of the most common media used to maintain physiological pH and osmolarity for biomedical applications in cell culture and protein chemistry [32,33]. Neutral pH (PBS 7.4) was chosen to mimic the physiologic conditions, whereas acidic conditions (PBS 5.4) were selected to approach the pathogenic conditions and the pH in some intracellular compartments. In situ AFM allows real-time monitoring of the degradation of individual MOF particles in terms of morphology, size variations, and mechanical properties with submicron resolution. Moreover, it has the advantage over other methods “bulk” such as PXRD, FT-IR spectroscopy, and porosimetry to enable individual particle characterization, giving insights on the homogeneity of the samples. Conversely, electron microscopy techniques allow investigating the morphological changes of individual particles during their degradation but obtaining three dimensional information is challenging and there are no possibilities to follow changes in mechanical properties. Therefore, in situ AFM was selected to investigate the erosion mechanism of MOFs (morphological, dimensional, and mechanical properties) in real time and with nanometric resolution.

So far, in situ AFM has been used mainly to investigate the mechanical properties of MOFs/composites, the crystal growth process, and the behavior of MOFs in the presence of other molecules [34,35,36,37,38]. Recently, two groups reported the in situ degradation of MOF microparticles. Hosono et al. synthesized flexible porous coordination polymer (PCP) crystals suitable for separation and storage applications and followed their dissolution in situ. Degradation occurred by a delamination process consisting in a layer-by-layer exfoliation of the framework [39]. In another study, a zinc-based imidazolate MOF, ZIF-8 (ZIF stands for Zeolitic Imidazolate Framework) was incubated in PBS, and its behavior was monitored by in situ AFM [9]. The morphology of the particles was immediately modified upon contact with PBS and the crystals totally dissolved in less than 15 min.

Microparticles of MIL-100(Fe) have been analyzed here by AFM for the first time to reveal their morphology, dimensional, and stiffness changes upon interaction with various media. Furthermore, liquid AFM enabled the in situ monitoring of the major parameters that drive MOF degradation: The composition and the pH of the medium in contact with the particles. In addition, the analysis of different individual crystals enabled assessing their homogeneity in terms of surface properties and degradation behavior.

To complete the study, both nano- and micron-sized MOFs were produced to assess the impact of the particle size on the degradation mechanism. In addition to the particle size, special attention was given to the quality of the MOF crystals since the synthetic route is known to affect the crystal quality (defects) and thus potentially impact the degradation mechanism. It was found indeed that the degradation kinetics depend on the synthesis method and the purification method. Three synthesis pathways were employed. First, hydrofluoric acid (HF) was used as mineralizing agent to promote MOF crystal growth [40] to obtain high-quality large particles named microMOFs (+). Despite the fact that it provided the best crystalline materials, HF is corrosive, difficult to handle, and several purification steps are needed. Alternative HF-free synthesis methods were employed leading to the formation of crystalline particles named microMOFs (−) [10] of around 20–70 microns and nanoMOFs of around 200 nm [10,16,17].

The surface topography and mechanical rigidity of the microMOFs in contact with various liquid media was studied by AFM showing that small defects onto the microMOFs (−) surface contributed to their degradation. On the opposite, well-defined crystalline planes were observed on the microMOFs (+), which were eventually eroded at a specific pH. Complementary insights were obtained by Scanning TEM with High Annular Dark Field Mode (STEM-HAADF) enabling to unravel the structure and composition of nanoMOFs upon degradation.

## 2. Materials and Methods

### 2.1. Materials and Reagents

The chemicals iron (0), 1,3,5-benzenetricarboxylic acid (BTC) and HF were purchased from Sigma-Aldrich (Saint-Quentin-Fallavier, France). Iron (III) chloride hexahydrate (98%) was purchased from Alfa Aesar (Kandel, Germany). Nitric acid, potassium hydroxide, absolute EtOH, and Dulbecco’s Phosphate Buffer Saline DPBS (1X, pH = 7.4) were purchased from Thermo Fischer Scientific (Les Ulis, France). DPBS contains 1.47 mM KH_2_PO_4_, 8.59 Na_2_HPO_4_·7H_2_O, 137 mM NaCl, and 2.66 mM KCl. Milli-Q water was obtained from a Millipore apparatus equipped with a 0.22 μm filter (18.2 MΩ cm). All reagents and solvents were used without further purification.

#### 2.1.1. Synthesis of microMOFs (−)

MicroMOFs were synthesized according to previous reported methods [10]. Briefly, a reaction mixture composed of 2.7 g iron (III) chloride hexahydrate (10.00 mmol) and 2.1 g trimesic acid (10.00 mmol) in 50 mL of water, was placed in a large Teflon bomb under magnetic stirring for 15 minutes. The Teflon reactor was encased in a metal bomb with controlled pressure, before being placed in an autoclave with a 1 hour heating ramp to 130 °C, and held at this temperature for 72  h. The product was cooled before being filtered and was then washed by heating under reflux, first in 700  mL of ethanol at 75  °C for 2 h and finally in 700 mL of water at 90  °C for 2  h. Finally, the resulting crystals were collected by filtration under vacuum and stored as powder after being dried in air.

#### 2.1.2. Synthesis of microMOFs (+)

Micron-sized MIL-100(Fe) crystals were successfully prepared by a hydrothermal method as previously reported [40]. This solid was isolated as a crystalline powder from a reaction mixture composed of 1.0 Fe^0^:0.66 1,3,5-BTC:2.0 HF:1.2 HNO_3_:280 H_2_O (1,3,5-BTC = benzene tricarboxylic or trimesic acid) that was held at 150 °C in a Teflon-lined autoclave for 6 days with an initial heating ramp of 12 h and a final cooling ramp of 24 h. The product was recovered by filtration and washed with deionized water.

#### 2.1.3. Synthesis of nanoMOFs

NanoMOFs were obtained by microwave-assisted hydrothermal synthesis as previously reported [10]. A mixture of iron chloride (8.97  mmol) and BTC (4.02  mmol) was placed in Teflon sealed autoclave reactors with 20  mL of deionized water and heated for 6  min at 130  °C under stirring. The applied power was 800 Watts (Mars-5, CEM, US) and pressure was fixed at 800 Watts. When temperature reached 90 °C, the reactors were placed in an ice bath for 10 min for stopping the nucleation. The resulting nanoMOFs were recovered by centrifugation (10,000 rpm, 15 min) and were washed with absolute ethanol 6 times to remove the residual non-reacted organic acid. A last centrifugation at 4000 rpm (2000× *g*) was performed during 1  min in absolute ethanol to sediment the largest particles and recover the supernatants as a suspension of monodisperse nanoparticles. NanoMOFs were stored in absolute ethanol until final use.

#### 2.1.4. Degradation of Nano and Micron Sized MOFs

For degradation studies, MIL-100(Fe) nanoMOFs were incubated under gentle stirring for 2 days in PBS 10 mM at 37 °C at a concentration of 0.25 mg/mL. Kinetic studies were carried out until achieving a total degradation of the frameworks. In the case of microMOFs, degradation studies lasted one month, as their kinetics were much slower. At each time point during degradation, nano/microMOFs were separated from their supernatant by centrifugation and were extensively washed with Milli-Q water, in order to stop the degradation process and to remove the remaining salts. Finally, the degraded particles were either kept in Milli-Q water or dried under vacuum according to their further characterization method.

### 2.2. Experimental Techniques

The changes in crystallinity upon degradation were performed by powder X-ray diffraction (PXRD) analysis in a D8 Advance Bruker Diffractometer in Debye–Scherrer geometry, in the 2θ range 2–40°. The diffractometer was equipped with a Ge (111) monochromator producing Cu Kα1 radiation (λ = 1.540598 Å) and a LynxEye detector. The size and the morphologies of the microparticles were determined by optical microscopy (Keyence VHX-7000). It was equipped with a motorized rotary turret of 4 high-resolution objectives covering a magnification of 20×–6000× and with a 4k camera. The microscope was equipped with an illumination system in reflection (dark and bright field) and an illumination in transmission. Keyence’s VHX software (7000, Kyoto, Japan) was used to reconstruct images without blurred areas and to perform measurements.

TEM/STEM studies were performed on a Titan Themis microscope (Palaiseau, France) corrected for spherical aberrations on the probe. The observations were made at 200 kV with a sufficiently low probe current, i.e., around 40 to 50 pA, so as not to degrade the sample and with an acquisition time of approximately 15 to 20 min. For the HAADF-STEM images acquisition, the half-angle of convergence of the probe was 17 mrad and the collection half-angle of the Annular Dark Field detector was 69 mrad (inner angle) and 200 mrad (outter angle).

For the TEM grid preparation, a 2 μL drop of the solution was placed on a 200 mesh copper grid covered with a pure carbon membrane (from Ted Pella).

To study the composition and the coordination of phosphates after degradation process, Infrared spectra were measured with a Nicolet iS5 FTIR ThermoFisher spectrometer (Courtaboeuf, France) between 400 and 4000 cm^–1^.

Porosity was evaluated by N_2_ sorption isotherms obtained at 77 K using a Micromeritics Tristar apparatus (Martignas, France). Before the analysis, around 30 mg of sample powder were activated by heating at 150 °C under secondary vacuum for 5 h.

Ligand release was studied by a reversed-phase High Performance Liquid Chromatography analysis (HPLC), as previously reported [41]. Waters Alliance e2695 Separations Module (Waters, Milford, MA) equipped with a UV-Vis detector Waters 2998 was used. A SunFire-C18 reverse-phase column (5 μm, 4.6 × 150 mm^2^, Waters) was employed. For the analysis of BTC, a mobile phase A consisting of a buffer solution (0.04 M, pH = 2.5) and a mobile phase B MeCN (50:50) were used. The injection volume was 50 µL and the detection wavelength was set at 225 nm. The column temperature was fixed at 25 °C [41].

For buffer preparation, NaH_2_PO_4_ (2.4 g, 0.02 mol) and Na_2_HPO_4_ (2.84 g, 0.02 mol) were dissolved in 1 L of Milli-Q water. The pH was then adjusted to 2.5 with H_3_PO_4_.

### 2.3. Atomic Force Microscopy

AFM works with a sharp probe tip attached to a cantilever, which deflects upon interaction with the surface according to Hooke’s law. A laser beam is used to detect the deflections of the cantilever induced by the force of tip–substrate interaction. Any movement of the cantilever changes the direction of the reflected beam and these changes are recorded by PSPD (position-sensitive photodiode), which makes it possible to obtain a topographic map of the sample under study. Force curves measure the sum of attractive and repulsive interactions between the cantilever and the sample as a function of distance and give valuable information about morphological deformations, dimensional changes (height measurements), and mechanical properties (stiffness) of the sample.

AFM studies were performed using a Bruker-JPK Instruments Nanowizard III (JPK Instruments AG, Berlin, Germany) and an electrochemical cell (ECCell Bruker-JPK Instruments). This equipment was associated with an inverted optical microscope (Axio Observer Z1 Zeiss, Göttingen, Germany). The entire system was placed on an anti-vibration table (Figure S1). AFM measurements were performed using a fast-speed approach/retract mode (Quantitative Imaging (QI) mode). At each pixel of the image, a complete force–distance curve, at a defined constant velocity, was acquired. The maximum force applied by the tip on the substrate was 2 nN, and the approach-retraction speed was constant at 150 μm·s^−1^. The data were acquired on 128 00D7 128 pixel images and processed by OriginPro (Origin Corporation, Northampton, MA, USA) and Matlab (MathWorks, Natick, MA, USA). Stiffness data were calculated from the slope of the force-distance curves at different points as selected from the obtained topographic images.

#### Sample Preparation for AFM

Borosilicate glass substrates coated by a thin layer of an indium-tin-oxide (ITO) were used for the deposition of the sample. The substrates were carefully rinsed with Milli-Q water and then dried with the flow of a dry dust suppressant (Jelt’R 99.9). One hundred and fifty microliters of microMOF sample were deposited to the ITO substrate and left to air dry for 10 min. The sample was then placed at the bottom of the liquid cell where it was washed six to eight times in a row with 600 μL of Milli-Q water to remove any particles not adhered to the substrate. First, studies were carried on in Milli-Q water. Then, investigations were performed in PBS. To do so, 600 μL of PBS pH = 7.4 were poured into the liquid cell and the MOF particles were visualized by an inverted optical microscope. When needed, the pH of the liquid medium was modified in situ by drop-wise addition of potassium hydroxide (KOH) 0.01M.

## 3. Results and Discussion

### 3.1. Synthesis and Characterization of MOF Crystals

**Figure 1:**
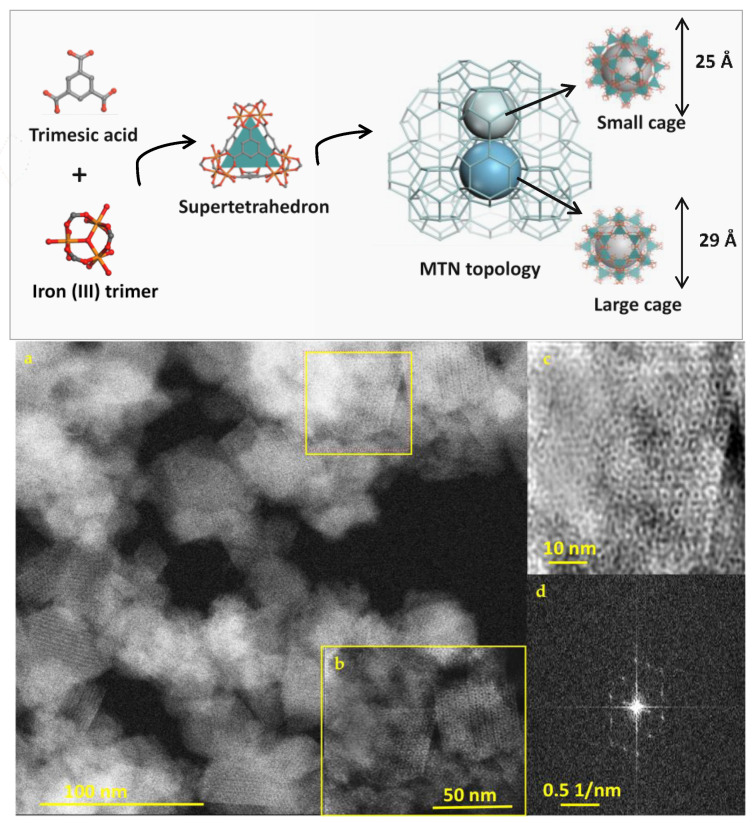
Upper panel: Schematic representation of the structure of MIL-100 (Fe) obtained by the self-assembly of iron supertetrahedra. Two cages 25 and 29 Å (small and large cage) are accessible through microporous pentagonal windows of 5 Å and microporous hexagonal windows of 8.6 Å. The structures were designed using Material Studio. Lower panel: Scanning TEM with High Annular Dark Field Mode (STEM-HAADF) images of MIL-100(Fe) nano Metal-Organic Frameworks (MOFs), (**a**) unprocessed image, (**b**) magnified image, (**c**) inverse FFT filtered STEM-HAADF image, and (**d**) FFT pattern which reveals the crystallinity of the particles. The ultrahigh resolution of STEM-HAADF shows the ordered porous structure of submicron particles.

MIL-100(Fe) is a highly crystalline porous material, resulting from the self-assembly of iron trimers and tricarboxylic ligands, leading to a 3D mesoporous structure with MTN topology. This structure has two types of cages (25 and 29 Å) accessible through microporous windows (5.5 and 8.6 Å) and exhibits Lewis acid sites able to bind various molecules (drugs, gases, vapors) [8,12,42]. Nano(micro)MOFs crystals were successfully synthesized, as determined by a series of complementary investigations. Their structure is schematized in Figure 1 (upper pannel).

First, STEM-HAADF, an ex situ advanced microscopic technique, was employed as a powerful tool for imaging local structures with atomic precision without damaging the samples. Highly ordered mesoporous cages were identified in agreement with the structure of MIL-100(Fe) by STEM-HAADF (Figure 1). Additionally, Fast Fourier Transform pattern (FFT) clearly confirms the crystalline character of the nanoMOFs.

However, STEM-HAADF cannot be used in the case of microMOFs due to their size above 20 microns. In contrast, these particles were visualized by optical microscopy (Figure S2). When stored in water, both types of microMOFs (+ and −) showed no detectable color or shape modifications.

PXRD patterns of synthesized micro and nanoMOFs were in agreement with previously reported data, confirming their crystalline structures [16,40] (Figure S3). Moreover, in line with published data [27,40], N_2_ sorption isotherms obtained at 77 K resulted in values of BET surface areas of 1700  ±  60 m^2^/g and 2080 ± 20 m^2^/g for microMOFs (−) and microMOFs (+), prepared as stated in introduction, by using HF or not, respectively. NanoMOFs surface area was 1900 ± 40 m^2^/g, in agreement with reported values [10,40] (Figure S4).

### 3.2. MOF Degradation in PBS

**Figure 2:**
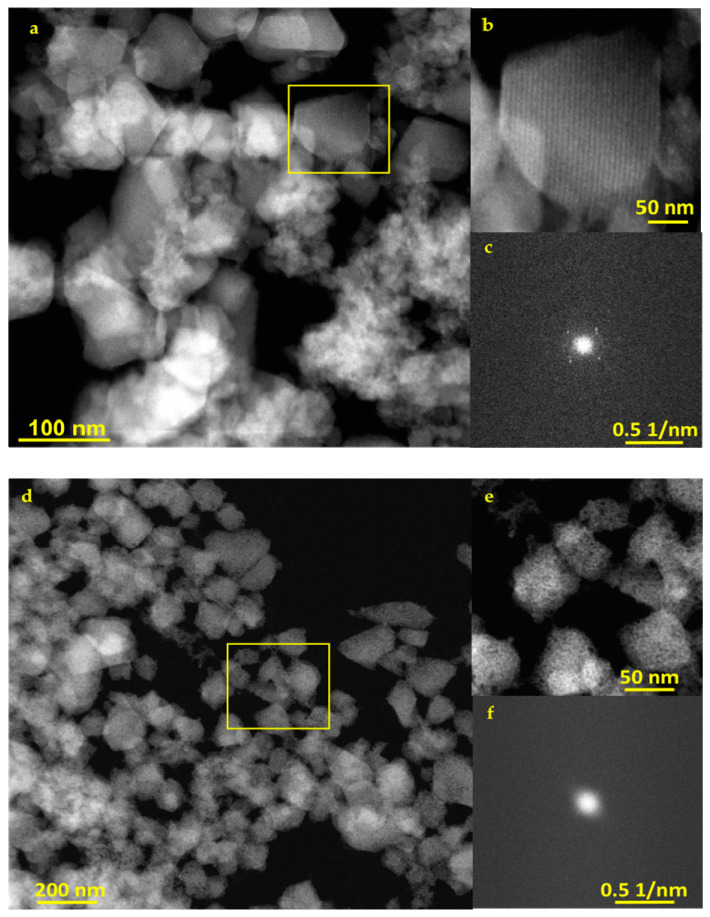
(**a**,**b**), STEM-HAADF images of degraded MIL-100(Fe) nanoMOFs in PBS (pH = 5.4) (**d**,**e**) in PBS (pH = 7.4) (**c**,**f**) FFT patterns of MIL-100(Fe) nanoMOFs in PBS pH = 5.4 and pH = 7.4, respectively.

In a comparative study, nanoMOFs were incubated under gentle stirring for 48 h in PBS (0.25 mg/mL) at pH = 5.4 and pH = 7.4 to study their degradation. First, they were visualized by STEM-HAADF to follow morphological changes. Interestingly, this technique evidenced that the well-formed nanoMOFs’ crystalline planes remained intact in PBS in acidic environment (pH = 5.4) (Figure 2a,b) but they were lost during degradation in PBS in neutral conditions (pH = 7.4) (Figure 2d,e).

More than 100 nanoMOF images were analyzed to measure the distance between their crystalline planes before any degradation, and the average value was found 4.1 ± 0.3 nm (Figure S5a). When degraded at pH 5.4, the distance remained exactly the same (4.1 ± 0.3 nm) (Figure S5b). This value corresponds to {111} crystal planes of MIL-100(Fe), the main planes with an inter-reticular distance of 4.23 nm. Moreover, FFT patterns indicate that the nanoMOFs partially maintained their crystallinity in acidic conditions, whereas they lost it entirely in neutral ones (Figure 2c,f).

According to HPLC investigations, the nanoMOFs progressively released their constitutive ligand in neutral PBS with 76 ± 7, 95 ± 8, and 100 ± 3 wt% after 1.5, 6, and 48 h, respectively, whereas in acidic conditions (pH = 5.4), the degradation kinetics was much slower and partial with 23 ± 1, 30 ± 1 and 38 ± 1 wt% loss at the same time points. Despite the erosion, the nanoMOFs’ mean diameters were only slightly modified, 140 ± 37 nm (intact) vs. 130 ± 11 nm and 125 ± 11 nm, upon 48 h degradation in PBS 5.4 and 7.4, respectively, as measured by STEM-HAADF. This moderate decrease in size is in agreement with previous reported data [10]. In contrast to STEM-HAADF, size measurements by DLS were not possible in reason of the nanoMOF aggregation, especially in PBS and whatever the pH.

In the case of microMOFs, optical microscopy was employed to study MOF size and morphology upon degradation. After incubation in PBS pH = 7.4, the microMOFs progressively changed their color. Specifically, in the case of microMOFs (−), it was previously reported the formation of an amorphous inorganic shell and an intact crystalline core, with no size and morphological changes [10]. Interestingly, microMOFs (+) also lost their initial color (from orange to transparent), but contrary to the microMOFs (−), color faded homogeneously in the whole structure (Figure S6). These changes could be related to evolutions of both MOFs’ composition and crystallinity upon degradation, the microMOFs (−), due to their higher defect content, being less homogenous than the microMOFs (+).

First, Fourier Transform Infrared spectroscopy (FTIR) demonstrated compositional changes attributed to the phosphates groups, which coordinated with the framework during degradation process. A typical example is given in Figure S7, showing the formation of a new (large) band at around 1050 cm^−1^, related to phosphate groups in the composition of the microMOFs upon their degradation in PBS. More specifically, this band corresponds to the asymmetrical P-O stretching [43,44]. Indeed, the degradation process follows a substitution mechanism. Firstly, the phosphates from the degradation media replace the bound water molecules, then they displace the organic linkers of the network which are released out.

Secondly, PXRD studies enabled to follow the evolution of the crystallinity of the particles upon degradation in PBS pH = 5.4 and pH = 7.4. After two days incubation in neutral PBS, nanoMOFs’ peaks disappeared entirely, leading to an amorphous material. Interestingly, in the more acidic medium the nanoMOFs kept a significant part of their crystallinity. The same studies were performed for microMOFs particles at different time points (from one to eight days) (Figure S8). It was found that after eight days of incubation in PBS (5.4 and 7.4) the characteristic Bragg peaks of microMOFs (+) disappeared, due to their degradation. The main ions that participate in the MOF erosion process are the phosphate ions, due to their highest tendency to form complexes with the iron sites of the framework. PBS is a buffer containing orthophosphoric acid (H_3_PO_4_), which exhibits three dissociation reactions.

$$H_{3}{\mathrm{PO}}_{4}\overset{{\mathrm{pKa}}_{1}}{⇌}H_{2}{\mathrm{PO}}_{4}{}^{-}{+\;H}^{+}\overset{{\mathrm{pKa}}_{2}}{⇌}{\mathrm{HPO}}_{4}{}^{2-}{+\;H}^{+}\overset{{\mathrm{pKa}}_{3}}{⇌}{\mathrm{PO}}_{4}{}^{3-}{+\;H}^{+\;}$$

In PBS at pH = 7.4, H_2_PO_4_^−^ and HPO_4_^2−^ species are in equilibrium at similar concentrations (5 mM). On the contrary, in the range of pH = 4–6, H_2_PO_4_^−^, anions are the predominant species compared to HPO_4_^2−^ (99:1 at pH 5.4). In both cases, phosphate ions diffuse into the MOFs and coordinate with the iron sites. It can therefore be hypothesized that the relative stability of the MOFs in acidic environment could be attributed to a predominant effect of H_2_PO_4_^−^ anions as compared to HPO_4_^2−^ ones. Yet, the stability of the framework also depends on the iron metal sites of the structure. The Pourbaix diagram of Fe in water shows that in neutral environment, the metal is less stable, compared to the acidic conditions (Figure S9). In the present case, studies were carried on in PBS where the formation of iron phosphates is a predominant mechanism [10]. Therefore, the erosion process is the result of the combined effects of the phosphate ions of the PBS and the stability of Fe within the MOF framework.

In a nutshell, whatever their size and preparation method, MOFs rapidly degraded in neutral PBS with variations in their optical properties, crystallinity, particle size, and composition. However, PXRD and IR spectroscopy are not quantitative methods leading only to partial conclusions. Of particular interest was the apparition of core-shell structures observed upon degradation in the case of microMOFs (−) but not with microMOFs (+). To gain deeper understanding on the degradation mechanism, AFM investigations were performed in situ with a series of microMOFs after optimization of their deposition onto the AFM ITO. However, liquid AFM is not adapted for nanoMOFs, which tend to detach from their support especially during degradation.

As a complement to characterization studies mentioned before, in situ AFM presents a particular interest, as it allowed visualizing local morphologies on the external surface of individual microcrystals, precisely calculating their dimensional changes and comparing their mechanical properties before and after degradation. It is also of great importance that these properties can be explored in real time, and not just on the final degraded product. Moreover, AFM allowed performing in situ changes of both composition and pH of the liquid media in contact with the MOFs, which is of man interest for the deeper understanding of their degradation mechanism. Based on these technical features, morphological, dimensional, and mechanical changes of the external surface of microMOFs (+) and microMOFs (−) were studied in water and in PBS.

### 3.3. In Situ AFM

#### 3.3.1. Morphology

**Figure 3:**
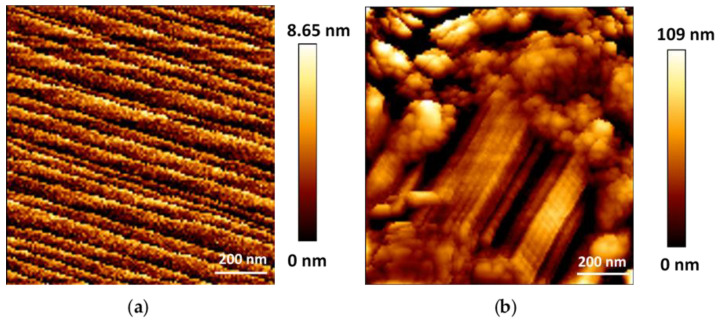
Topography of (**a**) microMOF (+) crystal and (**b**) a microMOF (−) crystal in water (128 pixels × 128 pixels). AFM mapping presents ordered crystalline planes typically observed for microMOFs (+) and some defects in the case of microMOFs (−).

To perform extensive in situ AFM studies and to be able to change the medium during the study, crystals’ immobilization on ITO substrate is crucial. Firstly, particles were deposited as ethanolic dispersions, and after solvent evaporation and extensive washing, a good adhesion of the microMOFs on the substrate was observed (Figure S10). Afterwards, distilled water was added in the liquid cell and the external surface of intact particles was observed by in situ AFM (Figure 3). Topographic mapping of microMOFs (+) presented regular formed crystalline planes in agreement with the previous results of STEM-HAADF for nanoMOFs (Figure 2a). The same experiments were carried out for microMOFs (−). Globally, the morphology of the external surface of microMOFs (−) also consists of crystalline regions (parallel lines, Figure 3b) but with a significant number of defects (cracks). Noticeably, these features remained unchanged upon storage in the aqueous medium, proving the stability of particles in water (Figure 3a,b).

**Figure 4:**
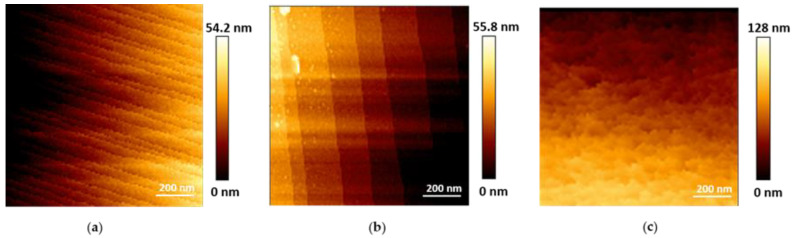
Topographic map of microMOFs (+) particles by in situ AFM (128 pixels × 128 pixels), in water (**a**), in PBS pH 5.4 (**b**) and in PBS pH 7.4 (**c**).

To degrade microMOFs, water was gently removed from the liquid cell and was replaced by PBS. Firstly, in situ AFM unravelled the surface structure of microMOFs (+) incubated in PBS at pH 5.4. Noteworthily, the crystalline planes already observed in water (Figure 4a) remained unchanged in an acidic environment (Figure 4b). In the next step, the pH of the degrading medium was changed in situ from acidic to neutral. Immediately after changing the pH to 7.4, erosion occurred, and irregular surfaces replaced the crystalline structures (Figure 4c).

**Figure 5:**
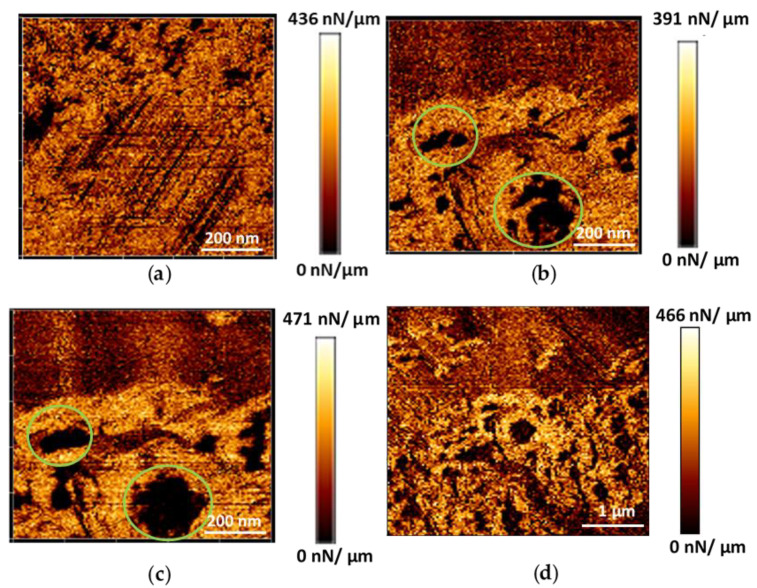
AFM images (stiffness map) (128 pixels × 128 pixels), of a microMOF (−) crystal in water at time 0 (**a**), in PBS pH = 7.4 at time 0 (**b**) and after 2 h at different magnifications (**c**,**d**). Defects that are progressively enlarging during degradation are surrounded with green circles.

In the case of microMOFs (−), degradation started from the existing defects of around 80–170 nm on their external surface (Figure 5b, green circles). Once the medium was changed (from water to PBS pH = 7.4) and the particles were in contact with the phosphate ions, an immediate expansion of these defects was observed, revealing the degradation of the crystals (Figure 5c,d).

In conclusion, microMOF (+) and (−) presented different morphologies strongly dependent on their initial defect content. The surface of microMOF (+) remained planar devoid of holes, whereas the initial defects on the external surface of microMOF (−) got larger and larger suggesting that these are the weakest regions to be degraded by phosphates from PBS, particularly at pH 7.4. Based on these findings, it was interesting to also investigate the dimensional changes of micron-sized particles upon degradation.

#### 3.3.2. Height Measurements

**Figure 6:**
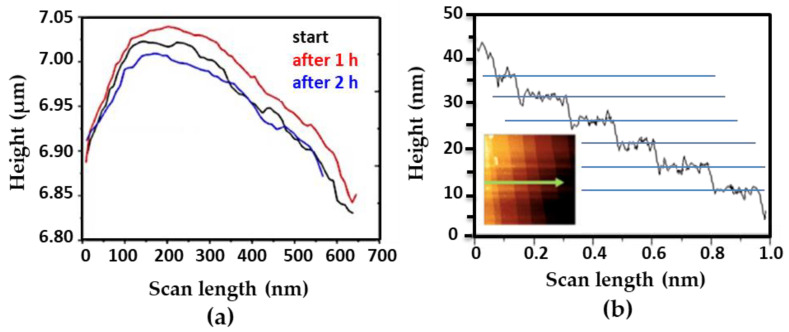
Typical height profiles of (**a**) a microMOF (−) crystal upon incubation in PBS pH = 7.4 up to 2 h and (**b**) a microMOF (+) crystal upon incubation in PBS pH = 5.4.

In contrast to electron microscopy techniques that generate images of a sample surface in two dimensions, AFM can measure the vertical dimension (z-direction), enabling quantitatively accurate calculations of sample’s height. To investigate dimensional changes of microMOFs upon degradation, height profiles were plotted. The typical profile is shown in Figure 6. In the case of microMOFs (−) (Figure 6a), regions were selected to include both crystalline regions and defects on the crystal. Surprisingly, the changes in height after 2 h of incubation in PBS at pH = 7.4, were less than 0.1% of the initial crystal size, indicating no dimensional changes. This result is in agreement with previous studies undertaken by Raman microscopy-spectroscopy showing no dimensional changes of microMOF (−) upon degradation in PBS at pH 7.4 [10]. This behaviour could be possibly attributed to the formation of a passivation layer. Following the height measurements for microMOFs (+), it was interesting to calculate the observed well defined crystal planes, in the presence of acidic PBS. The average value of plane distance was calculated and found at at 4.5 ± 0.3 nm. Remarkably, these values are in strong correlation with the results by STEM-HAADF for nanoMOFs, corresponding to the {111} crystal planes of MIL-100(Fe) of 4.23 nm.

As previously mentionned, the crystalline planes of microMOF (+) dissapear in PBS pH 7.4, leaving a rough irregular surface where height variations are difficult to be defined. In some rare cases, the formation of holes was observed (Figure S11). The height of the degraded region represented in this figure was calculated following the blue line. Interestingly, crystalline planes appeared at the edge of the observed hole at pH 5.4, whereas these were no longer observable on the surface of the crystal at pH = 7.4. However, the regularity in the spacing of these “steps” was lost: the differences in height from one plane to another are in most cases well over 4.5 ± 0.3 nm. The different heights of these “steps” are approximate multiples of this interplanar distance obtained at pH = 5.4.

In conclusion, in the case of crystalline microMOFs (−) with initial defects on their surface, as it was demonstrated above, particles did not present any significant dimensional change even after 2 h of degradation in PBS. In the case of well-crystallized microMOFs (+), the distances between the crystalline planes could be measured and remained constant upon incubation in acidic pH, where the crystals remained stable.

Although the crystal dimensions did not change, the progressive substitution of the organic ligand by phosphate ions led to a degraded product with different properties. In addition, we will show now that in situ AFM is a helpful tool to study the changes in the mechanical properties of microMOFs for both the degraded and non degraded regions of crystals, as well as their behavior after in situ change of the pH.

#### 3.3.3. Mechanical Properties

**Figure 7:**
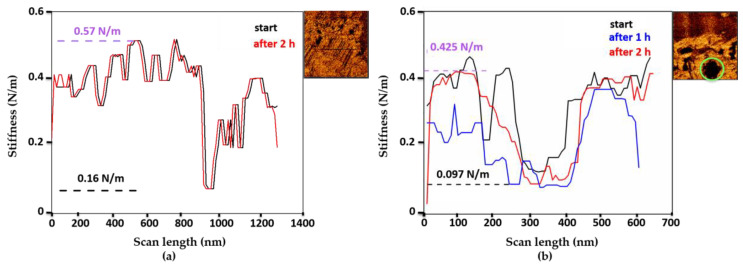
Stiffness profiles of a microMOF (−) particle upon incubation for 2 h in water (**a**) and in (**b**) PBS.

The mechanical properties were measured for intact and degraded microMOFs. Figure 7 represents these changes in water (a) and in PBS at pH 7.4 (b) upon incubation for 2 h. The mechanical properties did not change upon storage in water (Figure 7a), which is in line with the previous results (Figure 1 and Figure S5a) showing that the morphology was unaltered in water and that there was practically no ligand release. On the contrary, in PBS, in the presence of phosphates, degradation occurred, and the slope profile dramatically changed. More precisely, the degraded regions of the particles presented a significant decrease (a factor of 4.4) in terms of surface’s stiffness compared to the initially crystalline regions (Figure 7b). This indicates that the particles became more fragile upon contact with PBS. As expected, the stiffness of the natal default was low (green circles on Figure 7b), as a result of holes forming and growing.

In the case of microMOFs (+), topographic images (Figure 4) showed regular crystalline planes in acidic conditions, which were no more visible in neutral ones (after in situ modification of the pH). Before studying the mechanical properties of the particles at different pH conditions, the properties of two crystals incubated in acidic PBS were measured. Thus, modifications of surface properties upon in situ change of the pH can be valid for the intergity of the sample. To perform this comparison, mechanical properties for different regions of each crystal were also measured.

**Figure 8:**
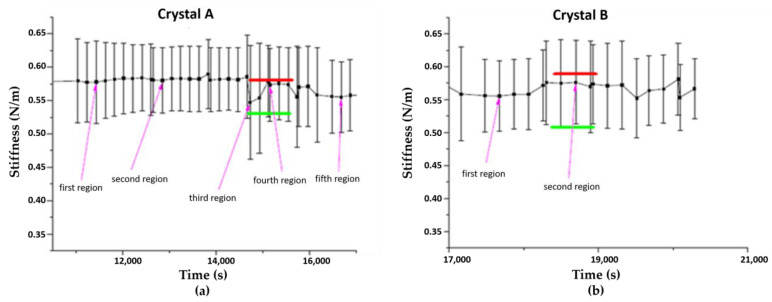
Comparison of the measured stiffnesses of different regions randomly chosen on two crystals named A (**a**) and B (**b**), in PBS at pH = 5.4. Results were averaged over regions of 1 μm^2^.

Firstly, five different regions of the same particle were chosen. The stiffness average was around 0.575 N/m, with a standard deviation of 0.075 N/m, indicating that the measured stiffness is similar regardless of the region of the crystal analyzed (Figure 8a). Two more regions of a second crystal gave the same result in terms of stiffness (Figure 8b). It can therefore be concluded that each individual crystal has a similar measured stiffness and that the whole sample has, in average, the same characteristics in terms of measured stiffness. As a result, in PBS at pH 5.4, each crystal has comparable rigidity values over its entire surface and each crystal is similar to the rest of the sample from which it is derived. It is possible at any time to analyze a given region of a given crystal to study its rigidity to obtain significant results for the whole sample.

**Figure 9:**
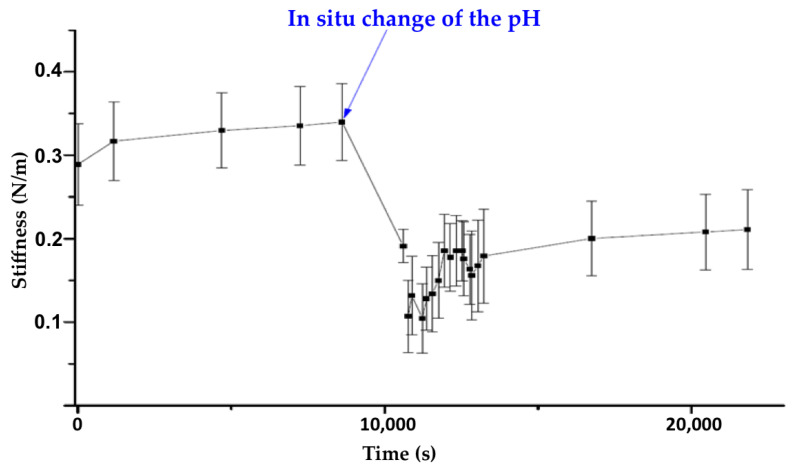
Stiffness profile of the evolution of the measured stiffness of a microMOF (+) particle following the addition of KOH.

Therefore, we analyzed the surface stiffness of a crystal following an in situ pH change from 5.4 to 7.4. Immediately after the addition of a few drops of KOH, the stiffness values dropped rapidly (Figure 9) from 0.325 N/m (with a standard deviation of 0.05 N/m) at pH 5.4 to 0.20 N/m (with a standard deviation of 0.05 N/m) at pH 7.4. These results are in agreement with the loss of the crystalline planes and the formation of a degraded product.

## 4. Conclusions

Depending on their synthetic methods, MOFs present different BET surface areas and degrees of crystallinity. AFM studies showed that the surface of large crystals has well-defined crystalline planes and might possess small crystal defects where degradation preferentially occurs, propagating then through the MOF material. The MOFs eroded mostly at neutral pH and not under acidic conditions. Noticeably, the MOF samples maintained their 3D structure without collapsing nor presenting dimensional changes, possibly because of the formation of a passivation layer. In the case of well-crystallized MOFs, degradation occurred homogeneously over the entire surface of the material with the disappearance of the crystalline planes at the surface. Degradation occurred homogeneously also in nanoMOFs, which showed homogeneous compositions and morphologies over time. Initially very well crystallized, the nanoMOFs degraded without noticeable size modification. This study highlights the fast response of MOF materials to changes in the composition of the aqueous media they are in contact with. In contrast to microMOFs, nanoMOFs develop much larger surface areas where interactions take place. NanoMOFs are essentially developed for intravenous administration, also for pulmonary, ocular, or transdermal applications, whereas microMOFs could find applications as implants or for oral delivery of drugs. In all cases, the understanding of the changes in composition and mechanical properties is important. AFM studies showed that MOFs become very fragile upon degradation, but remarkably, without losing their initial shape. The MOF methodology presented here can open perspectives to study MOF degradation in more complex media, i.e., in the presence of proteins or serum. The results presented here are of utmost importance for a deeper understanding of the fate of MIL-100(Fe) MOFs administered in vivo where they experience rapid changes both in the pH and the composition of their surrounding media.

## Supplementary Materials

The following are available online at https://www.mdpi.com/2079-4991/11/3/722/s1, Figure S1: Apparatus of AFM, Figure S2: Optical images of intact microMOFs (+). Figure S3: PXRD patterns (λ_Cu_ = 1.5406Å) of intact nanoMOFs and microMOFs (+) and their simulated pattern. Figure S4: N_2_ adsorption isotherms of intact nanoMOFs (blue) and of microMOFs (−)(red) at 77 K (P_0_ = 1 atm), Figure S5: Crystalline ordered planes of a) intact microMOFs (+) particles and b) upon incubation in acidic PBS by STEM-HAADF microscopy, Figure S6: Optical images of synthesized microMOFs (+) before (a) and after (b) degradation in neutral PBS for 1 month, Figure S7: FTIR spectra of microMOFs (+) upon incubation in PBS 10 mM pH = 5.4 and pH = 7.4. Figure S8: PXRD patterns of degraded nanoMOFs and microMOFs (+), Figure S9: Pourbaix diagram of iron. Figure S10: MOF crystal structure and cantilever-tip assembly visualized by inverted optical microscopy. Figure S11: Topographic image of a microMOF (+) degraded particle in PBS 7.4 and its plotted height profile as a function of sample’s distance representing the loss of crystalline planes.
