# Supplementary material for: Degradation Mechanism of Porous Metal-Organic Frameworks by In Situ Atomic Force Microscopy
Source: Nanomaterials (Basel). 2021 Mar 13;11(3):722. doi: 10.3390/nano11030722 (PMC8001454; doi:10.3390/nano11030722)
Supplement: Supplementary file 1 [file nanomaterials-11-00722-s001.pdf]

nanomaterials

MDPI

Supplementary Materials

Degradation Mechanism of Porous Metal-Organic Frameworks by In Situ Atomic Force Microscopy

Ioanna Christodoulou [1,2], Tom Bourguignon [1], Xue Li [1], Gilles Patriarche [3], Christian Serre [2], Christian Marlière [4] and Ruxandra Gref [1,*]

1 Institute of Molecular Sciences, UMR CNRS 8214, Université Paris-Saclay, 91400 Orsay, France; ioanna.christodoulou@universite-paris-saclay.fr (I.C.); tom.bourguignon@universite-paris-saclay.fr (T.B.); xue.li@universite-paris-saclay.fr (X.L.)
2 Institut des Matériaux Poreux de Paris, UMR 8004, Ecole Normale Supérieure, ESPCI Paris, CNRS, PSL University, 75005 Paris, France; christian.serre@ens.psl.eu
3 Center for Nanoscience and Nanotechnology, UMR 9001, CNRS, Université Paris Saclay, Palaiseau, France; gilles.patriarche@c2n.upsaclay.fr
4 Laboratoire de Physique des Solides, UMR CNRS 8502, Université Paris Saclay, 91400 Orsay, France; christian.marliere@universite-paris-saclay.fr
* Correspondence: ruxandra.gref@universite-paris-saclay.fr

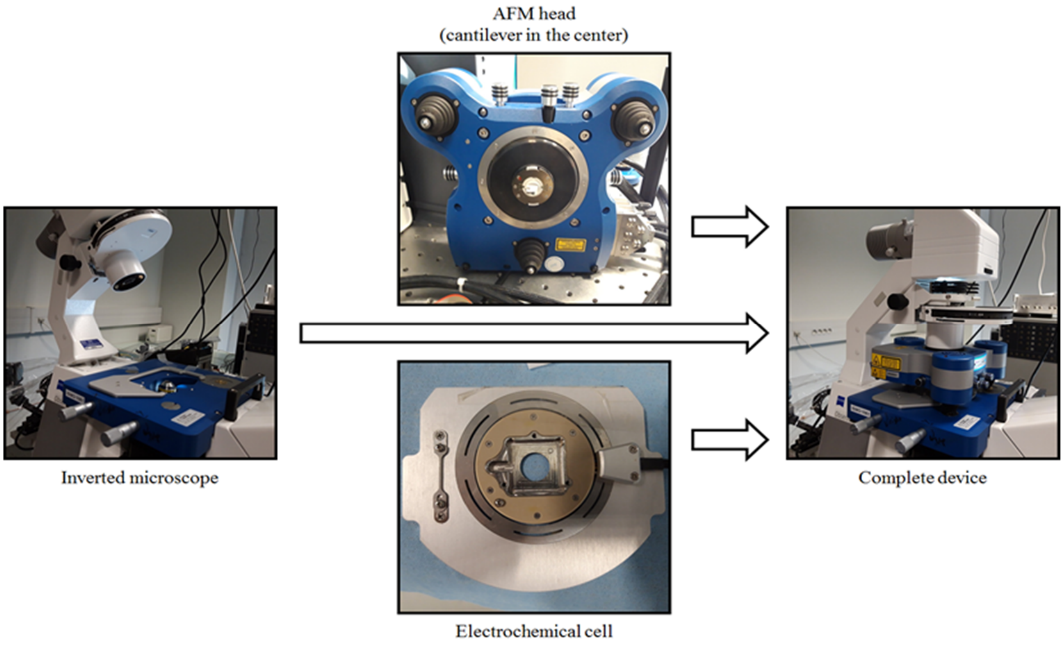

Figure S1. Apparatus used for AFM studies.

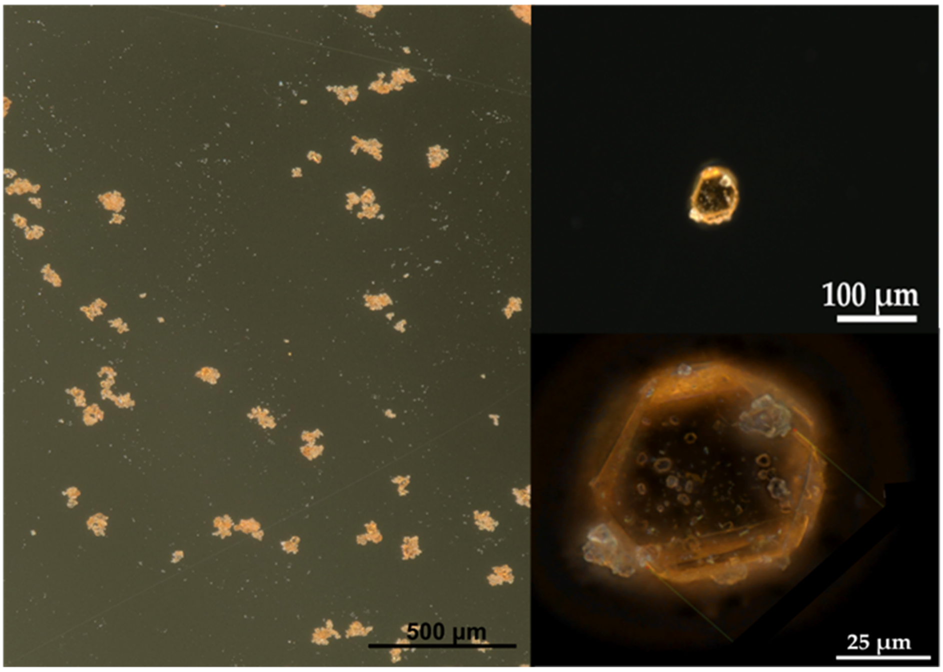

Figure S2. Optical images of intact microMOFs (+). The Fe-based particles present a typical intense orange color.

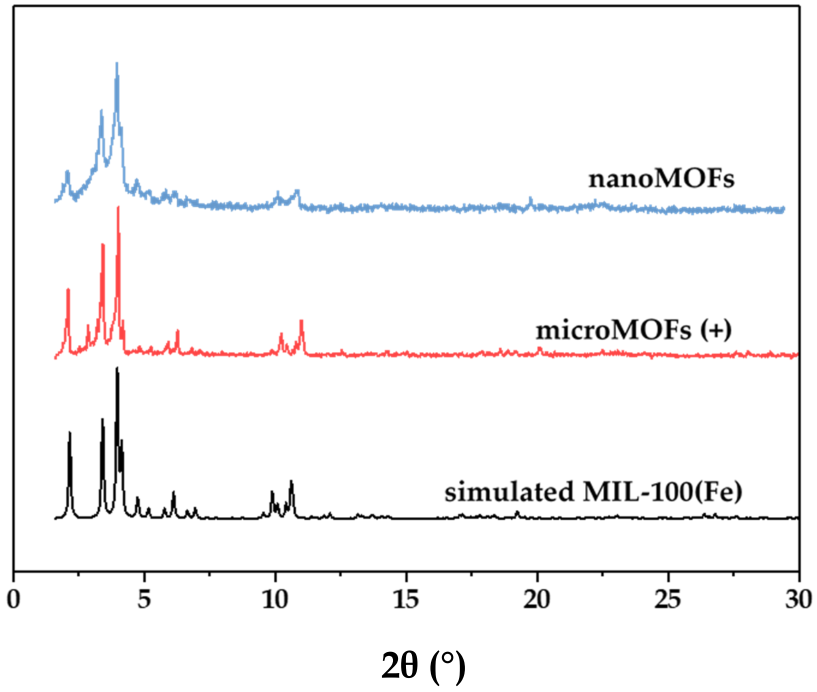

Figure S3. PXRD patterns (λ_{Cu}=1.5406Å) of intact nanoMOFs and microMOFs (+) and their simulated pattern.

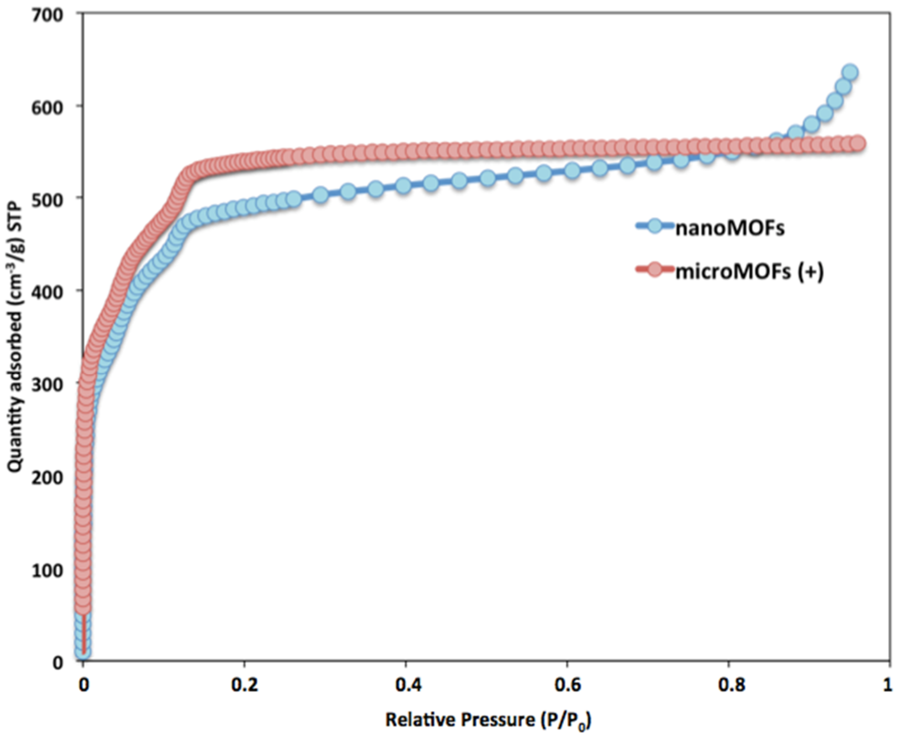

Figure S4. N_2 adsorption isotherms of intact nanoMOFs (blue) and of microMOFs (−) (red) at 77 K (P_0 = 1 atm).

(a)

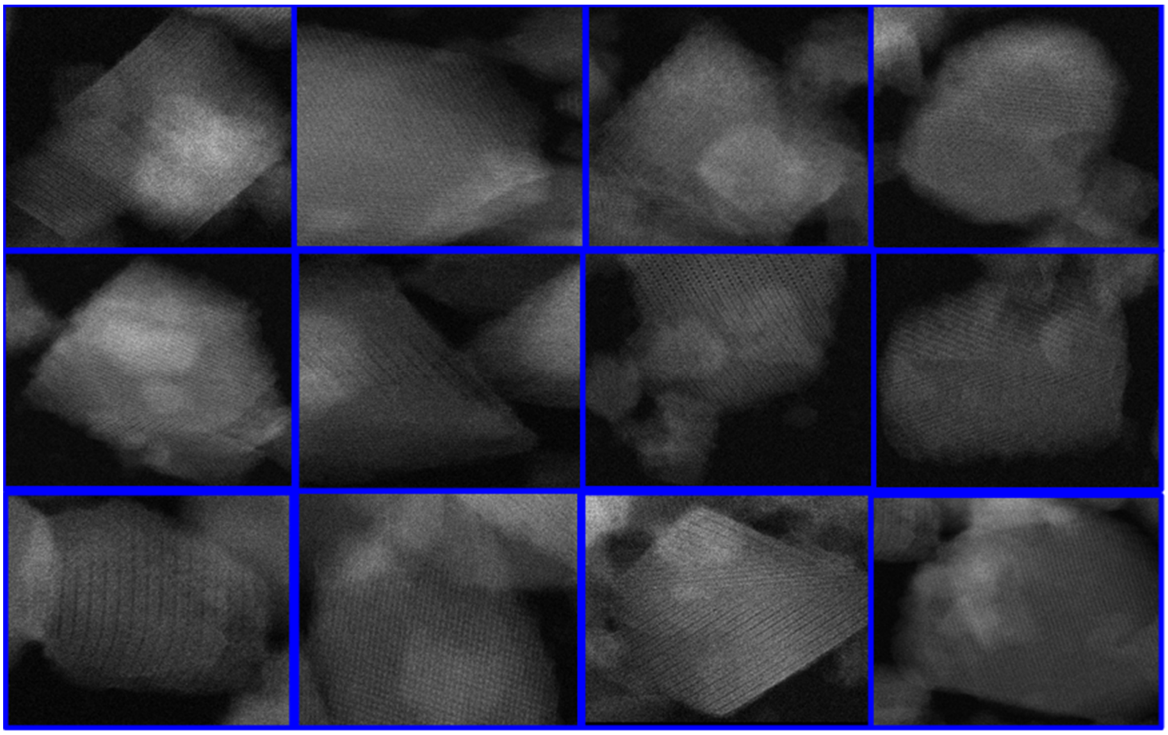

(b)

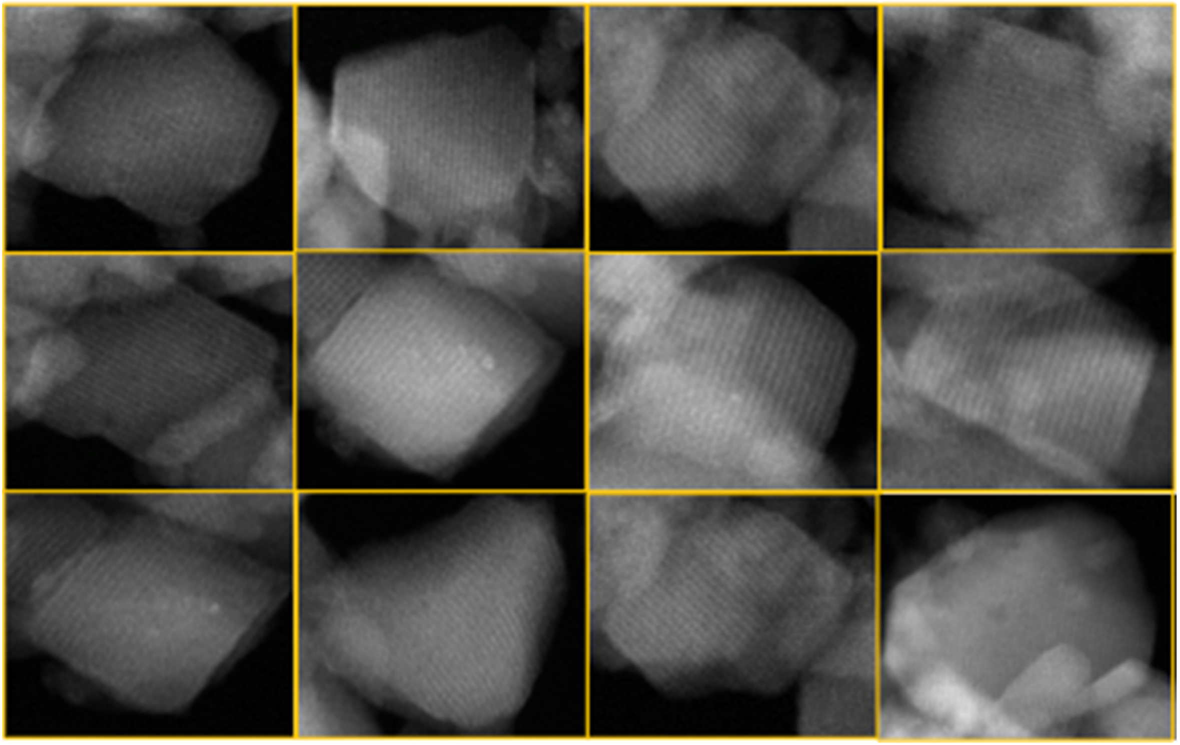

Figure S5. STEM-HAADF of MIL-100(Fe) (**a**) intact and (**b**) after 48 hours incubation in PBS 10 mM pH = 5.4. The microscopic images present well formed crystalline planes of various nanoMOFs that remain in acidic conditions.

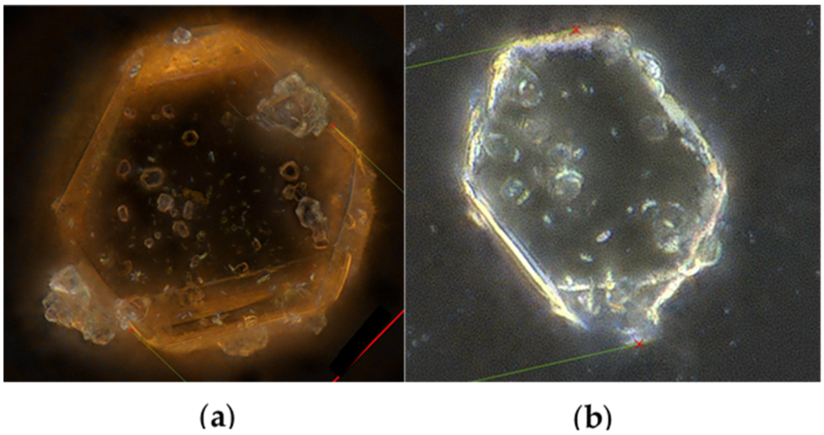

Figure S6. Optical images of synthesized microMOFs (+) before (**a**) and after (**b**) degradation under physiological conditions for 1 month.

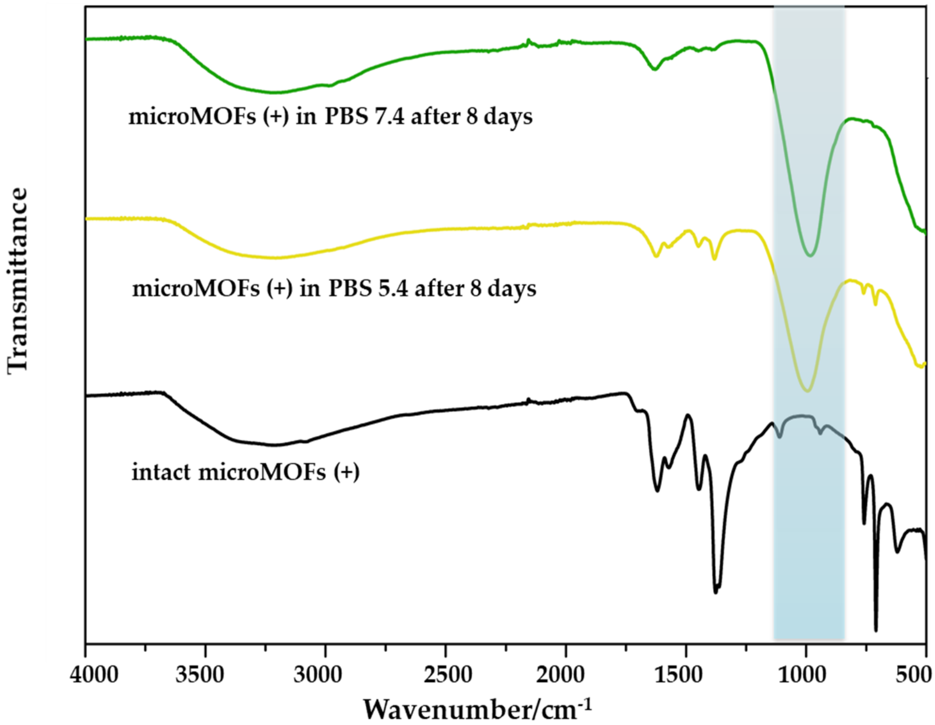

Figure S7. FTIR spectra of microMOFs (+) after 8 days incubation in PBS 10 mM pH = 5.4 (yellow) and in PBS 10 mM pH = 7.4 (green).

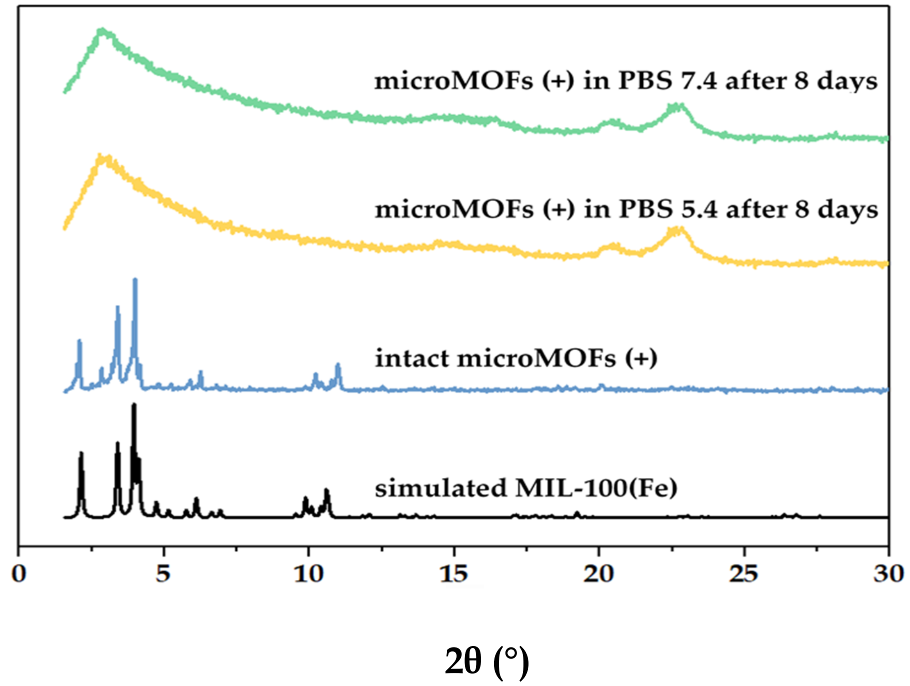

(a)

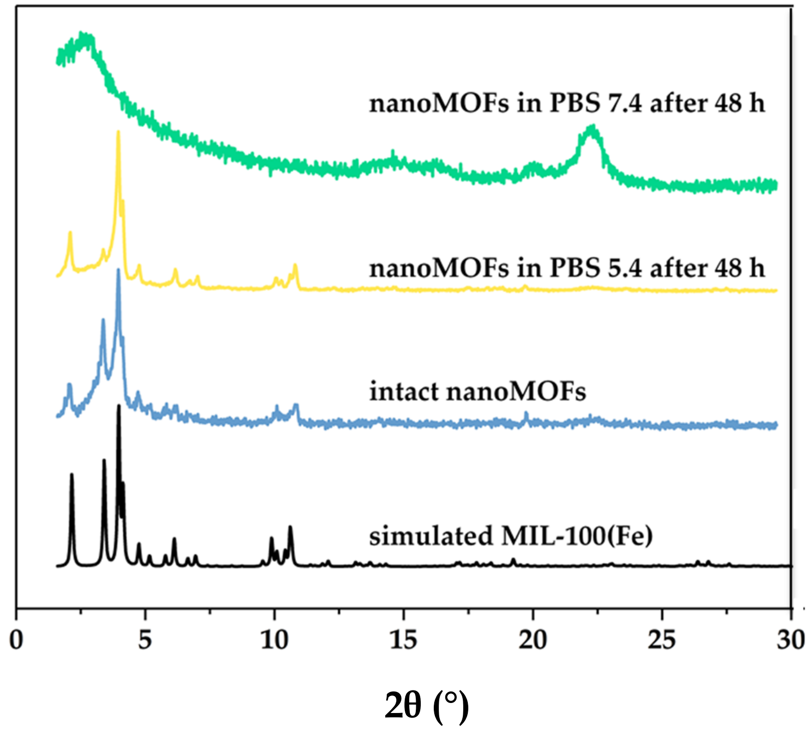

(b)

Figure S8. PXRD patterns of MIL-100(Fe) (**a**) microMOFs and (**b**) nanoMOFs before and after degradation in PBS pH=5.4 and 7.4.

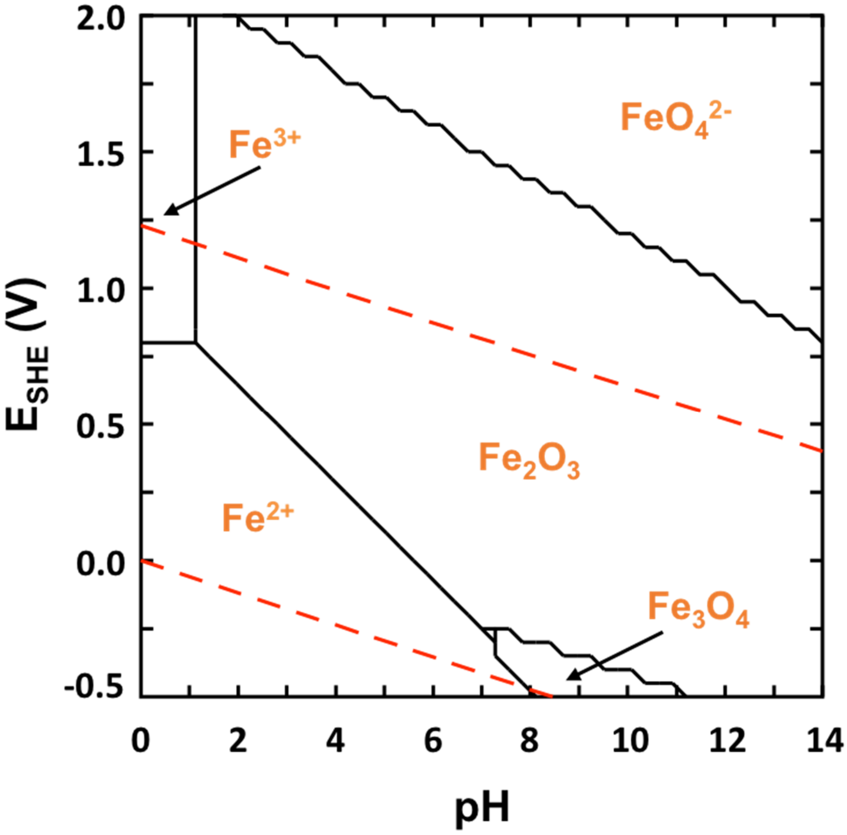

Figure S9. Pourbaix diagram of iron calculated for ionic concentrations of 1.0 mM at 25 °C, using the Hydra/Medousa software. Red dashed lines represent the redox couples O_2/H_2O and H_2O/H_2.

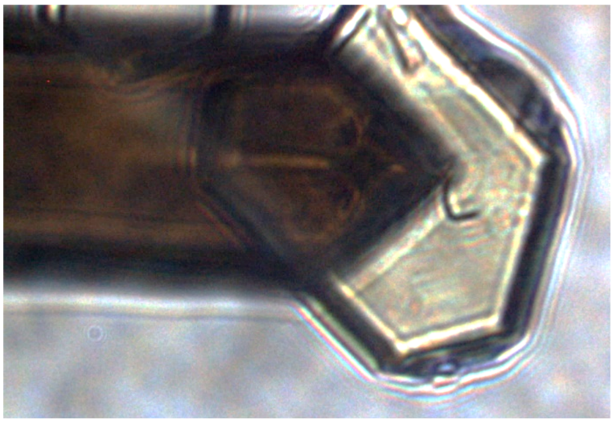

Figure S10. MOF crystal structure and cantilever-tip assembly visualized by inverted optical microscopy.

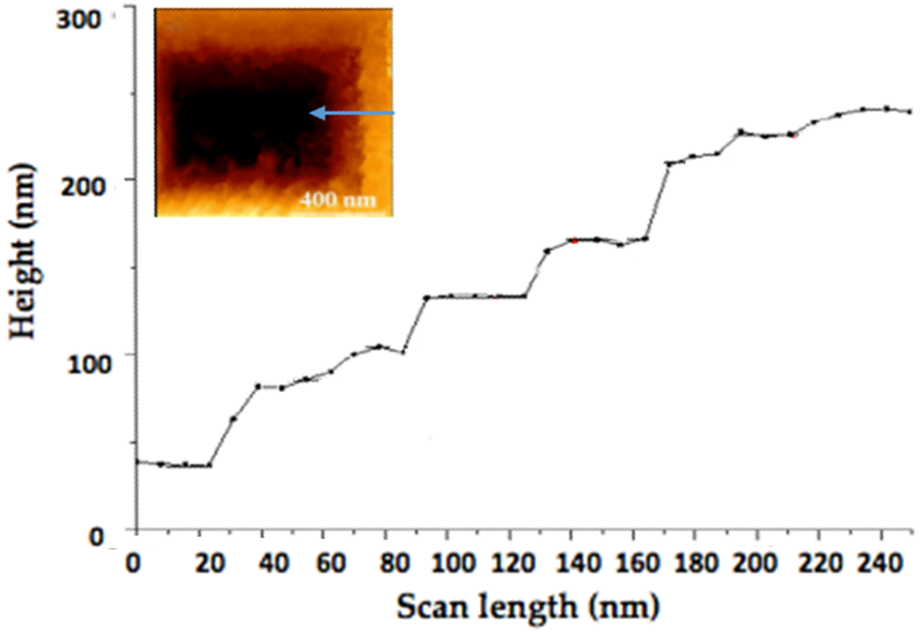

Figure S11. Topographic image of a microMOF (+) degraded particle in PBS 7.4 10 mM and its plotted height profile as a function of sample's distance representing the loss of crystalline planes.

© 2021 by the authors. Licensee MDPI, Basel, Switzerland. This article is an open access article distributed under the terms and conditions of the Creative Commons Attribution (CC BY) license (http://creativecommons.org/licenses/by/4.0/).
